# A pen in the liver

**DOI:** 10.1016/j.radcr.2022.07.096

**Published:** 2022-08-17

**Authors:** Jenifer Barrie, Dileep N. Lobo

**Affiliations:** a Gastrointestinal Surgery, Nottingham Digestive Diseases Centre and National Institute for Health Research (NIHR) Nottingham Biomedical Research Centre, Nottingham University Hospitals NHS Trust and University of Nottingham, Queen's Medical Centre, Nottingham NG7 2UH, UK; b MRC Versus Arthritis Centre for Musculoskeletal Ageing Research, School of Life Sciences, University of Nottingham, Queen's Medical Centre, Nottingham, UK

**Keywords:** Ingested foreign bodies, CT scan, Imaging, Hepatoduonenal fistula, Surgery

## Abstract

A 24-year-old woman with anxiety, depression, and emotionally unstable personality disorder was referred to a tertiary center 2 weeks after ingesting multiple foreign bodies. She had undergone a laparoscopic cholecystectomy and a laparotomy for extraction of ingested foreign bodies several years ago. A sagittal CT scan view showed a ballpen and a hair clip in the stomach. A coronal view demonstrated that a second ballpen had penetrated the duodenal wall to enter the liver parenchyma. There was no free intraperitoneal air or fluid or evidence of abscess formation. At laparotomy, a toothbrush, a broken spoon and a ballpen were extracted from the stomach via an anterior gastrotomy. The duodenum was adherent to the liver but the second ballpen had migrated into the distal duodenum, with the tip in the proximal jejunum. This was extracted via an enterotomy and the fistula was not interfered with. The enterotomy and gastrotomy were closed with 3-0 polydioxanone sutures. The hair clip had passed spontaneously and was not detected on intraoperative fluoroscopy. She made an uneventful recovery and postoperative liver function tests remained in the normal range. This is only the fourth reported case of a pen fistulizing between the upper gastrointestinal tract and the liver.

## Introduction

Ingestion of foreign bodies can occur either accidentally or deliberately. It has been estimated that while up to 80% of ingested foreign bodies pass through the gastrointestinal tract spontaneously, 10%-20% necessitate endoscopic removal and less than 1% need surgical treatment [1]. Complications can occur in up to 5% of patients [1] and include laceration, perforation, bleeding, migration, obstruction, fistulization and abscess formation. Foreign bodies migrating from the gastrointestinal tract to the liver are rare and when reported, they usually consist of fish or chicken bones, needles and toothpicks [2]. They commonly present with liver abscesses and patients may not provide a history of foreign body ingestion [2]. We present a case of a pen migrating from the duodenum to the liver causing fistulization without free perforation or abscess formation.

## Case report

**Fig. 1:**
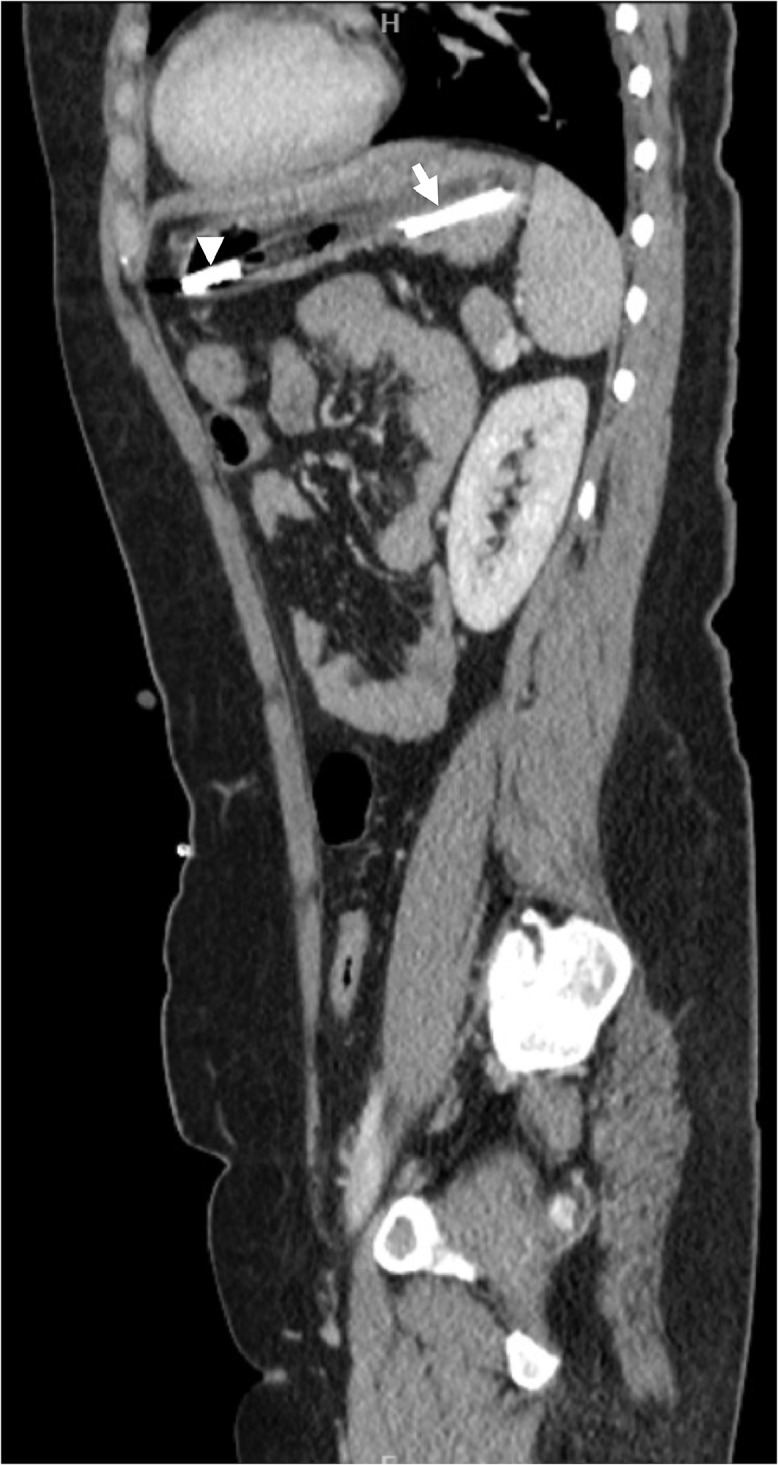
Sagittal CT scan view of the abdomen showing a hair clip (white arrowhead) and a ballpen (white arrow) in the stomach.

**Fig. 2:**
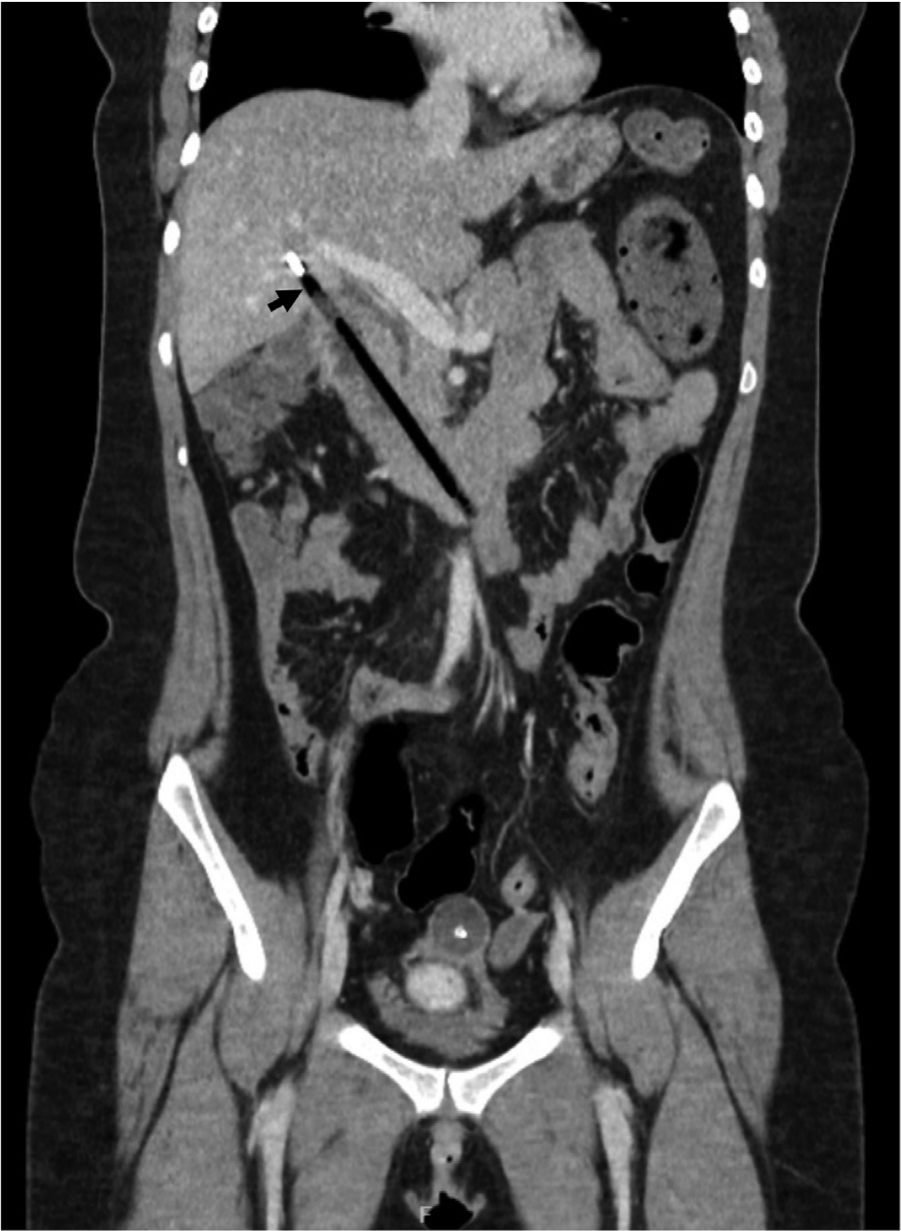
A coronal CT scan view of the abdomen showing fistulization between the duodenum and the hepatic parenchyma with a ballpen in the fistula (black arrow). There was no evidence of free gas or fluid in the peritoneal cavity.

A 24-year-old woman with anxiety, depression, and emotionally unstable personality disorder who was detained under Section 3 of the Mental Health Act (for medical treatment) was referred to a tertiary center 2 weeks after ingesting multiple foreign bodies. She had undergone a laparoscopic cholecystectomy and a laparotomy for extraction of ingested foreign bodies several years ago. She also had Fowler's syndrome (urethral sphincter relaxation disorder) for which a suprapubic catheter was inserted. She admitted to having swallowed 2 pens, a toothbrush and a broken plastic spoon. She complained of intermittent pain in the right upper quadrant of the abdomen but was otherwise well. Clinical examination was unremarkable and blood tests, including liver function tests, were normal. A sagittal CT scan view (Fig. 1) showed a ballpen and a hair clip in the stomach. A coronal CT scan view demonstrated that a second ballpen had penetrated the duodenal wall to enter the hepatic parenchyma (Fig. 2). There was no free intraperitoneal air or fluid or evidence of abscess formation.

**Fig. 3:**
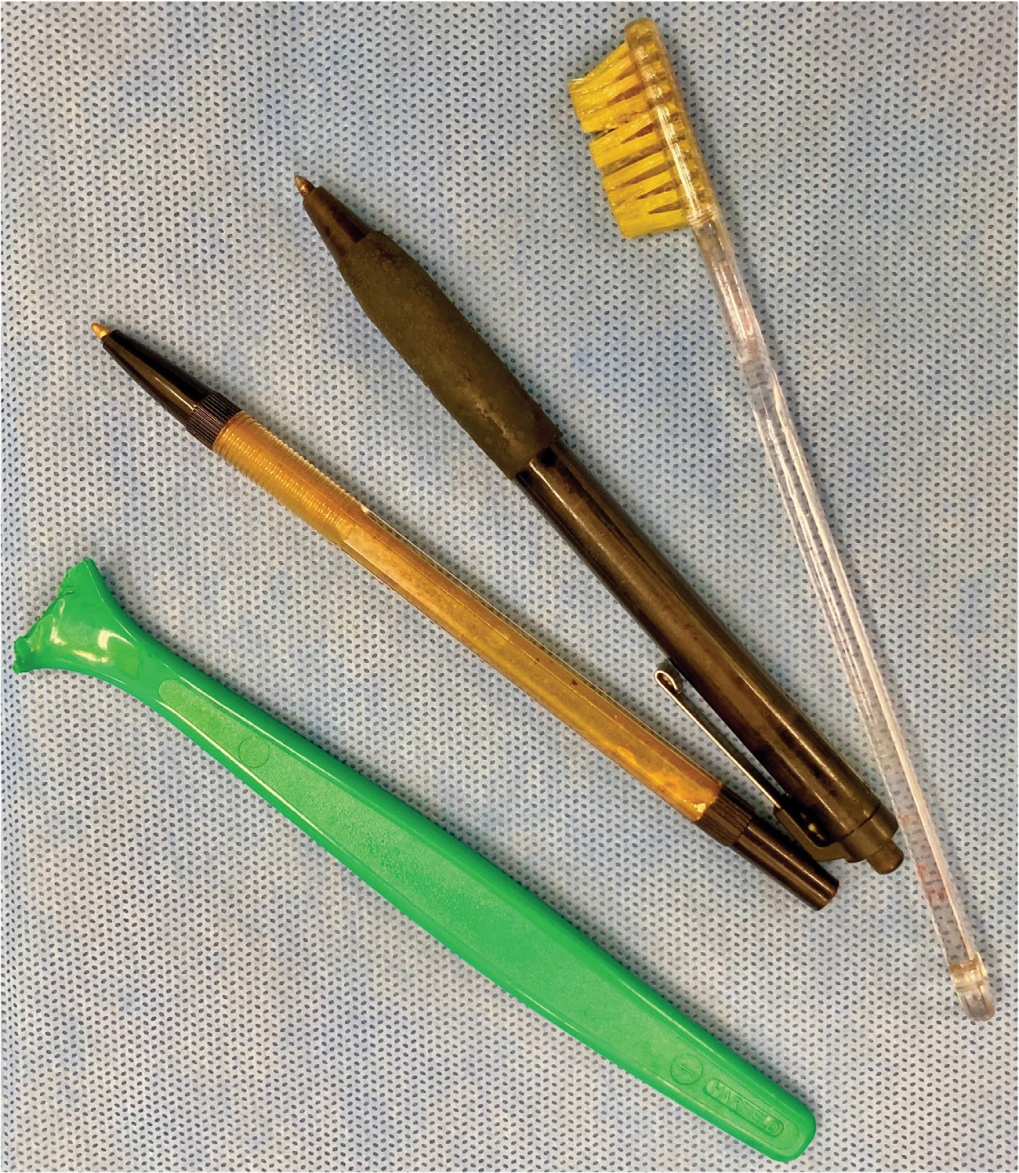
Two ballpens, a broken spoon and a toothbrush were extracted via a gastrotomy and an enterotomy.

At laparotomy, a toothbrush, a broken spoon and a ballpen (Fig. 3) were extracted from the stomach via an anterior gastrotomy. The duodenum was adherent to the liver but the second ballpen had migrated into the distal duodenum, with the tip in the proximal jejunum. This was extracted via an enterotomy and the fistula was not interfered with. The enterotomy and gastrotomy were closed with 3-0 polydioxanone sutures. The hair clip had passed spontaneously and was not detected on intraoperative fluoroscopy. She made an uneventful recovery and postoperative liver function tests remained in the normal range. She was discharged back to the mental health facility where she was being treated.

## Discussion

The migration of ingested foreign bodies from the gastrointestinal tract to the liver is rare and a systematic review performed in 2010 revealed that there were only 59 reported cases causing liver abscesses [2]. Although ingested pens may cause gastrointestinal perforations occasionally, there are only 3 previously reported cases of pens fistulizing from the duodenum or stomach into the liver [3–5].

The first reported case was that of a 43-year-old man who presented with fever, leucocytosis and anemia [3]. CT scan revealed a large right-sided hepatic abscess and pneumobilia. There were multiple foreign bodies in the gastrointestinal tract with the nibs of 2 pens lying in the duodenum. The liver abscess was drained percutaneously and treated with antibiotics. Endoscopy was performed and foreign bodies, including an embedded pen, were removed from the stomach and duodenum. The second pen was thought to have passed spontaneously. A 25-year-old woman presented one week after ingesting a pen [4]. The pen was visible in the stomach on abdominal X-ray. No free air was noted in the abdomen. Gastroscopy revealed that the pen was lying within the pylorus, but the proximal end appeared to have passed through the lesser curvature of the stomach. No attempt at extraction was made. A subsequent CT scan showed that the tip of the pen had perforated the lesser curvature of the stomach, resting at the inferior border of the left lobe of the liver. There was a small amount of surrounding free air, and some free fluid. At laparotomy, the pen was found to have perforated through the stomach into the left lobe of the liver. The pen was extracted, and the gastric defect closed. An incidental pericardial effusion was found in a 57-year-old woman [5]. She developed fever 7 months later and a CT scan showed that an ingested pen had perforated through the first part of the duodenum and caused an abscess in the left lobe of the liver. The pericardial effusion was thought to be secondary to local inflammation. Laparotomy was performed with drainage of the liver abscess, removal of the foreign body and repair of the duodenum.

A recent systematic review has revealed that deliberate foreign body ingestion occurs in up to 92% of all adult patients presenting with foreign body ingestion and up to 85% have a history of psychiatric illness [6]. The review also suggested that 84% of patients presenting with deliberate ingestion of foreign bodies had done so previously [6]. Psychiatric conditions commonly associated with deliberate ingestion of foreign bodies include psychosis, malingering or the Munchausen syndrome, obsessive compulsive disorder and personality disorder [6]. Including the present case, 3 [3,5] of the 4 cases of ingested pens fistulizing between the upper gastrointestinal tract and the liver had a history of mental illness. These included anxiety, depression and emotionally unstable personality disorder (present case), schizophrenia, alcohol misuse disorder, self-mutilation, obsessive water drinking and pica [3], and schizoaffective disorder and substance use disorder [5]. No mention of mental illness was made in the fourth case [4].

The 2016 European Society of Gastrointestinal Endoscopy guidelines for removal of foreign bodies in the upper gastrointestinal tract [7] do not recommend radiological evaluation for patients with non-bony food bolus impaction without complications. Plain radiography is recommended if ingestion of radiopaque objects is suspected or the type of object is unknown. CT scans should be performed in all patients with suspected perforation or other complications that may necessitate surgery.

While the vast majority of ingested foreign bodies pass spontaneously, some need endoscopic retrieval. The European Society of Gastrointestinal Endoscopy guidelines recommend urgent (within 24 hours) therapeutic upper gastrointestinal endoscopy for foreign bodies in the stomach such as sharp-pointed objects, magnets, batteries and large/long objects and nonurgent (within 72 hours) therapeutic endoscopy for medium-sized blunt foreign bodies in the stomach [7].

However, for complex cases, surgical intervention may be necessary. Three of the 4 cases of pens fistulizing from the upper gastrointestinal tract to the liver needed surgery, with one being managed with percutaneous drainage of the liver abscess and endoscopic extraction of the foreign body [3]. In these cases, principles of surgery include removal of the foreign bodies, repair of free perforations, drainage of abscesses, and treatment of contamination if present. Surgical disconnection and repair of the fistula is not essential as it may increase the complexity of the operation and increase morbidity.

A thorough psychiatric history is mandatory in patients presenting with deliberate ingestion of foreign bodies and early psychiatric evaluation must be sought in new patients. Hesitation on the part of the patient to report self-injurious behavior due to fear or shame, motivations for secondary gain or suicide intent can lead to worse clinical outcomes [6]. Occasionally, there may be a delay in carrying out medical procedures because of an inability to obtain informed consent. Subsequent management of patients with deliberate ingestion of foreign bodies and mental illness includes maintaining a safe environment, managing the sequelae of the most recent episode, and attempting to prevent future episodes by using pharmacological and psychosocial management strategies [6].

## Patient consent

The patient has provided written informed consent for this submission. A copy is available upon request.
